# Privacy-Preserving in Healthcare Blockchain Systems Based on Lightweight Message Sharing

**DOI:** 10.3390/s20071898

**Published:** 2020-03-29

**Authors:** Junsong Fu, Na Wang, Yuanyuan Cai

**Affiliations:** 1 School of Cyberspace Security and National Engineering Lab for Mobile Network Technologies, Beijing University of Posts and Telecommunications, Beijing 100876, China; fujs@bupt.edu.cn; 2 School of Computer Science, Beijing University of Posts and Telecommunications, Beijing 100876, China; 3 National Engineering Laboratory for Agri-Product Quality Traceability and Beijing Key Laboratory of Big Data Technology for Food Safety, Beijing Technology and Business University, Beijing 100048, China; caiyuanyuan@btbu.edu.cn

**Keywords:** privacy-preserving, electronic medical records, lightweight message sharing, healthcare blockchain system

## Abstract

Electronic medical records (EMRs) are extremely important for patients’ treatment, doctors’ diagnoses, and medical technology development. In recent years, the distributed healthcare blockchain system has been researched for solving the information isolated island problem in centralized healthcare service systems. However, there still exists a series of important problems such as the patients’ sensitive information security, cross-institutional data sharing, medical quality, and efficiency. In this paper, we establish a lightweight privacy-preserving mechanism for a healthcare blockchain system. First, we apply an interleaving encoder to encrypt the original EMRs. This can hide the sensitive information of EMRs to protect the patient’s privacy security. Second, a (t,n)-threshold lightweight message sharing scheme is presented. The EMRs are mapped to *n* different short shares, and it can be reconstructed by at least *t* shares. The EMR shares rather than the original EMRs are stored in the blockchain nodes. This can guarantee high security for EMR sharing and improve the data reconstruction efficiency. Third, the indexes of the stored EMR shares are employed to generate blocks that are chained together and finally form a blockchain. The authorized data users or institutions can recover an EMR by requesting at least *t* shares of the EMR from the blockchain nodes. In this way, the healthcare blockchain system can not only facilitate the cross-institution sharing process, but also provide proper protections for the EMRs. The security proof and analysis indicate that the proposed scheme can protect the privacy and security of patients’ medical information. The simulation results show that our proposed scheme is more efficient than similar literature in terms of energy consumption and storage space, and the healthcare blockchain system is more stable with the proposed message sharing scheme.

## 1. Introduction

Electronic medical records (EMRs) play an important role in people’s healthcare [1]. With the increasing demand of cross-institution sharing, massive data processing, and medical quality improving, the current centralized healthcare service system cannot keep up with the rapid development of modern healthcare [2,3]. In recent years, blockchain technology [4,5] has been applied to solve the weak points in traditional systems, and hence, the distributed healthcare blockchain system appears [6,7]. In order to protect user privacy and defend sensitive information exposure, EMRs should be encrypted before uploading to the healthcare blockchain system. Traditional data encryption schemes are stuck in the high complexity and inefficient data processing. Thus, exploring privacy-preserving approaches based on a lightweight message sharing scheme is of paramount importance. Massive medical data processing in the healthcare blockchain system is particularly challenging as it is extremely difficult to meet all the requirements of performance, system security, and efficiency.

In traditional healthcare service systems, the centralized organizations control the whole system, and all the EMRs are locally stored. In this case, the adversaries can tamper with the historical records for their benefit regardless of the patients’ lawful rights and interests. EMRs contain the sensitive information of the patient and medical institution, such as the patient’s name, ID number, telephone number, medical institution name, etc. The centralized cloud storage structure cannot provide full protection for EMRs. Moreover, the integrity of the EMRs can be also easily destroyed by the inevitable software/hardware failures and human errors in the cloud. In addition, different medical institutions are loath to share their data due to the privacy concerns and competitive advantages [8,9]. The consistency and interoperability of the different types of data from different medical institutions are big problems for data sharing [10].

Recently, outsourcing the local EMRs to the public cloud has attracted more and more attention. This is reasonable considering that compared with local data management systems, the cloud service is more cost-effective, green, and extensible. However, similar to the centralized healthcare service systems, the cloud-based methods also have to establish sharing channels through different public cloud platforms for different data users and institutions [11,12]. Apparently, these methods cannot break away from the drawbacks of the centralized systems. In conclusion, although it can facilitate EMRs cross-institutional sharing compared with the traditional healthcare service systems, the information redundancy always makes the data exchanging process inefficient [5].

The healthcare blockchain system presents a new possibility to solve the information isolated island problem in traditional centralized systems [6,7,13,14]. Similar to the Bitcoin system [4], the blockchain provides a public, auditable, and inalterable ledger, which can guarantee the data security and transparency for transactions’ implementation. The patients can obtain continuous and trackable treatment by freely accessing the healthcare information of their EMRs from the healthcare blockchain system. The cross-institutional sharing of EMRs will be easy with many medical institutions joining in this healthcare blockchain system, so the patient do not need to construct many EMRs at different medical institutions. However, the integrated EMRs data are always too large, which will lead the system to be more bloated and inefficient. This can be explained by the fact that each EMR needs to be stored in each node of the blockchain, and hence, the total needed storage space is extremely large. Considering the great amount of the EMRs, the storage efficiency and data transmission efficiency need to be further improved.

It can be observed from the above schemes that there exists a common problem in centralized healthcare service systems and distributed healthcare blockchain systems that the data storage and data sharing processing are not efficient with the massive data. Fortunately, lightweight message sharing can solve this problem perfectly. Data storage in a distributed manner between different medical institutions is an extremely important field, and the security and integrity of EMRs also cannot be ignored. In this paper, we introduce the secret sharing technique to the blockchain, and this improves the data storage efficiency, data transmission efficiency, and the security of the EMRs. Specifically, we establish a lightweight privacy-preserving mechanism for the distributed healthcare blockchain system.

In order to protect the data privacy and improve the system efficiency, we first design an interleaving encoding algorithm and propose a lightweight message sharing scheme. The interleaving encoder divides the original EMRs into *t* pieces, which can hide the sensitive information of EMRs by destroying the semantic meanings. The message sharing scheme is a (t,n)-threshold scheme, which constructs the former *t* pieces into *n* shares for storage. Then, the original EMRs can be reconstructed with only t(1<t≤n) shares. Therefore, this message storage and sharing scheme is lightweight with shorter shares and an efficient reconstruction process. After constructing the shares of the new generated EMRs, all the shares are transmitted to different nodes on the blockchain. Note that each share of an EMR is only stored in one blockchain node. This is totally different from the traditional blockchains in which the data are repeatedly stored in all the blockchain nodes. Another challenge is how to retrieve the EMRs for the data users based on the blockchain. In our scheme, all the nodes can generate blocks and append the blocks of the chain similar to existing blockchains. However, in our blockchain, the shares of EMRs are not stored in the block, and instead, the hash values of the EMR identifiers that are related to the shares are stored in the blocks. In the retrieval process, the data users can first search the public blocks to locate the nodes where the shares of an EMR are stored and then request the shares from the nodes. Once at least *t* shares are received, the data user can at last recover the original EMR. Security analysis and simulation results show that the proposed scheme can not only make the EMRs data complete and secure, but also make the processes of data storage and sharing more efficient.

The main contributions of this paper are summarized as follows:

- We propose a more lightweight and efficient privacy-preserving mechanism for EMRs. The EMRs can be securely and freely exchanged among different medical institutions through the distributed healthcare blockchain system.
- We apply the interleaving encoder technique to the privacy-preserving mechanism. It can protect the sensitive information of the patient and medical institution by destroying the semantic meanings of the original EMRs.
- We propose a new lightweight (t,n)-threshold message sharing scheme to improve the efficiency of data processing in the healthcare blockchain system. We also present a detail security analysis of the EMRs’ privacy protection protocol, which shows the correctness and security of the proposed scheme.
- We give the performance evaluation and analysis of the proposed scheme. The simulation results show that it can provide strong protection of the patient’s and medical institution’s privacy. Meanwhile, the proposed scheme is more efficient than similar literature with respect to the energy consumption and storage space.

The rest of this paper is organized as follows: In Section 2, some related works about the healthcare service system, healthcare blockchain, and message sharing scheme are given. In Section 3, the lightweight privacy-preserving mechanism with the interleaving encoder algorithm and (t,n)-threshold message sharing scheme is proposed. In Section 4, the security analysis of the proposed scheme is presented. In Section 5, we give the performance evaluation and analysis of the proposed scheme. In the end, the conclusions are given in Section 6.

## 2. Related Works

### 2.1. Healthcare Service Systems

**Figure 1:**
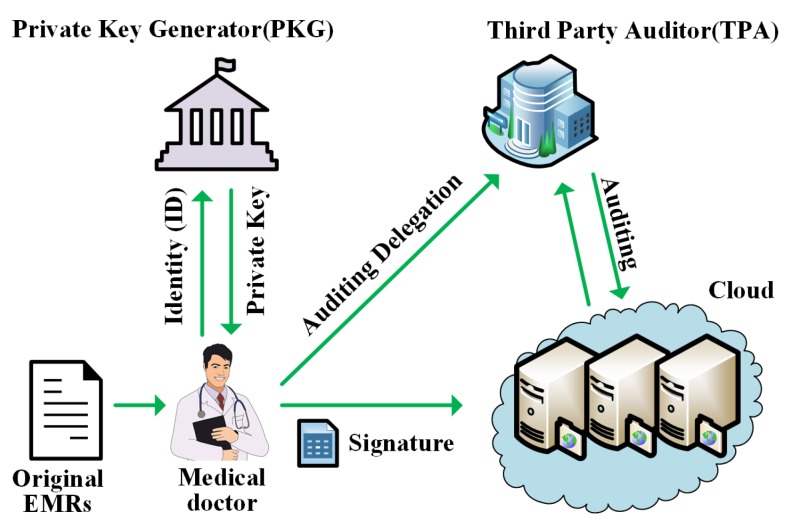
An example of a healthcare service system.

EMRs represent the most important information for diagnosis and treatment in healthcare, which generally contain the sensitive information of the patients and the medical institutions. Figure 1 shows an example of the traditional centralized healthcare service system [2,3]. The medical doctor, private key generator (PKG), third party auditor (TPA), and the cloud storage server are the main components of the system, and they all have rights to access the EMRs. However, the most important members, i.e., the general patients, cannot freely access their EMRs, especially when a medical tangle occurs. The original EMRs are produced by the medical doctor and uploaded to the cloud with his/her signature. Then, they can be collected and researched to seek more suitable therapies and improve the medical care level. However, as the data in the cloud always need to be frequently distributed and shared, the sensitive information of EMRs may be easily exposed to multiple users such as insurance companies, researchers, and others. This poses a major challenge for the sensitive information security.

The PKG and TPA are the representative parts of the centralized healthcare service system. As shown in Figure 1, the PKG generates the private key for the medical doctor to sign the original EMRs or the patients to check their healthcare data. Although the personal EMRs data can be protected by signatures, they can be easily tampered with and deleted since the PKG takes control of all the users’ private keys. The TPA is responsible for auditing the healthcare data. In this centralized system, the medical institutions take control of the whole system, which may easily cause data deletion, tampering, and other problems once the PKG or TPA becomes malicious. Unfortunately, these centralized systems will bring many new problems, such as the hurdles of the agreement of the supporting technical architecture and infrastructure, the security risk, and the operational control of data. Therefore, the inevitable software bugs, hardware faults, and human errors in the systems can easily lead to data corruption and loss.

### 2.2. Blockchain for Electronic Medical Records Sharing

The blockchain has been a research hotspot in recent years, which is a promising technology to solve the problems in the centralized systems. Bitcoin was the first application of the blockchain technology, which constructs a peer-to-peer electronic cash system [4]. Proof of work (PoW) [5] is a consistency algorithm used in Bitcoin and most modern blockchain-enabled systems to realize the distributed consensus among unfamiliar users. The signature algorithm [15,16] is also needed to protect the users’ privacy and transaction security. The blockchain is usually considered as a public, decentralized, distributed, and reliable database with high Byzantine fault tolerance [17] and used in finance, cloud computing, IoT systems, and other applications.

Cross-institutional sharing of healthcare data is pressing, but unprocurable with current centralized systems, and many distributed healthcare service systems based on blockchain technology have been given more research in recent years [6,7,10,13,14,18,19,20,21,22]. Theses literature works provide a significant exploration of data sharing among different traditional healthcare service system, and the blockchain technology always serves as a distributed ledger to record and store the EMRs. Although these distributed healthcare blockchain systems provide a public platform for the free exchange of EMRs, the privacy-preserving protocol and message sharing scheme for data security also should be given more consideration.

### 2.3. Message Sharing Schemes

Message sharing schemes [23,24,25,26] are good methods for EMRs’ cross-institutional exchange, which can guarantee the messages’ security and integrity through the delivery processes between the users, the healthcare blockchain system, and the consumer. The first message sharing scheme was proposed by Shamir [24], which was constructed with a threshold access structure in which an original message can be divided into *n* shares and recovered by at least t(1<t≤n) shares. From then on, many classical (t,n)-threshold message sharing schemes have been proposed [24,27,28]. Pang et al. [27] presented another (t,n)-threshold message sharing scheme in a simple manner, but the length of the shares was too excessive. Then, a lightweight message sharing scheme was given for source-location privacy protection in wireless sensor networks, which presented a more promising method to manage data with short length shares and transmitting them securely in an energy-efficient manner [28].

There are also some secure data sharing schemes that have been proposed to strengthen the privacy security of EMRs. The scheme in [11] was a collaborative message sharing protocol, and it provided inter-organizational sharing of the healthcare data. Yang et al. [12] presented an IoT-based storage system for healthcare big data privacy-preserving with self-adaptive access control. An identity-based integrity auditing and data sharing scheme was proposed in [29], and it also could hide the sensitive information for privacy protection. However, the above three schemes had some problems, such as low efficiency and weak robustness, by executing on centralized cloud storage.

## 3. Lightweight Privacy-Preserving Mechanism of EMRs Based on Blockchain and Secret Sharing

**Figure 2:**
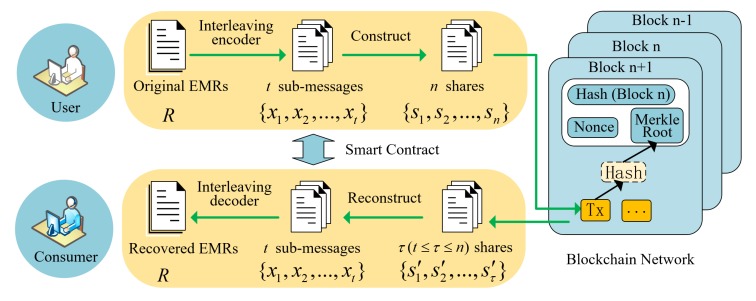
The framework of the lightweight privacy-preserving mechanism in the healthcare blockchain system.

In this section, we propose the lightweight privacy-preserving mechanism for EMRs based on secret sharing in the distributed healthcare blockchain system. The framework is shown in Figure 2, and the main terms are listed in Table 1. In order to improve the security and scalability of the EMR sharing system, we designed a lightweight (t,n)-threshold message sharing scheme for the privacy-preserving of healthcare blockchain system. Meanwhile, we discuss how to store and search the shares of EMRs. The framework mainly was comprised of two main parts: creation and storage of the shares of EMRs and the recovery and use of EMRs. The detailed steps of the protocol are shown as follows.

### 3.1. Creation and Storage of the Shares of EMRs

The patient and medical doctor are the main participants who create the original EMRs *R*. In order to hide the sensitive information and protect the whole original EMRs, they upload and store the shares rather than the plaintext EMRs in the blockchain nodes. The steps of constructing and storing the shares are presented in the following.

**Figure 3:**
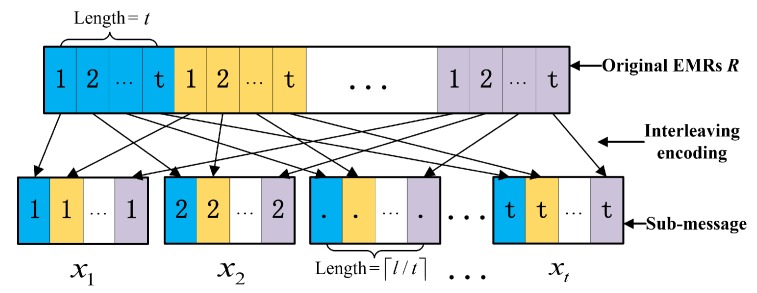
The principle of the interleaving encoder.

EMR interleaving encoding: First and foremost, the original EMRs *R* will be encoded into a series of sub-messages by an interleaving encoder as shown in Figure 3. We first divide the *l*-bit original EMRs *R* into ⌈l/t⌉(1<t≤n) groups, and each group has *t* bits. Here, we always add (t−(lmodt))-bit 0 at the end of the *l*-bit string *R*. Then, we encode them into *t* sub-messages $\left\{x_{1},x_{2},\ldots ,x_{t}\right\}$ with the length of ⌈l/t⌉. By splitting and recombining the original information, the adversary can only obtain insignificant messages even if they can obtain several shares, because the interleaving encoder has destroyed the semantic meanings of the shares.

Construction of the EMR shares: In this step, the encoded EMRs $\left\{x_{1},x_{2},\ldots ,x_{t}\right\}$ will be constructed into *n* different shares $s_{i}\left(i=1,2,\ldots ,n\right)$ based on Equation (1).

(1) $$s_{i}=\left\{\begin{array}{c} s_{1}+\cdots +s_{i-1}+ix_{i}+x_{i+1}+\cdots +x_{t}\;\mathrm{mod}\;p,\mathrm{if}\;1\leq i\leq t\\ \frac{s_{1}}{i-t+1}\;+\;\frac{s_{2}}{i-t+2}\;+\;\cdots \;+\;\frac{s_{t}}{i-t+t}\;\mathrm{mod}\;p,\mathrm{if}\;t<i\leq n \end{array}\right.$$

Here, *p* is defined as the largest prime number that is not greater than $2^{\left\lceil l/t\right\rceil }$. The size of $s_{i}$ is always smaller than ⌈l/t⌉ of *p*. As the size ⌈l/t⌉ of shares is much smaller than the size *l* of the original message, it will make the message sharing scheme more lightweight and greatly improve the efficiency of data processing. The EMRs’ construction encrypts *t* sub-messages into *n* shares, which can further strengthen the protection of user privacy.

Storage of the shares in blockchain nodes: Through the interleaving encoder and construction of the shares, the original EMRs are encrypted into *n* shares. Then, the shares will be sent to different blockchain nodes, and they are stored locally in the nodes. Meanwhile, the indexes of these shares will be uploaded into the healthcare blockchain system. Similar to the transaction verification in Bitcoin [4,30], all the indexes of the shares and the corresponding identifiers of the block nodes are combined together and broadcast to the whole healthcare blockchain network, i.e., all the blockchain nodes, for verification.

EMRs’ confirmation and generating a new block: When one node obtains the rights for creating a new block by the consensus mechanism, the indexes of EMR shares and the information about where they are stored in the nodes will be recorded and stored in the healthcare blockchain system. Considering that the information stored in the blocks cannot be modified, the blockchain nodes cannot deny that the corresponding EMR shares are stored by them.

### 3.2. Recovery and Use of EMRs

When an authorized data user wants to search an EMR, he/she first needs to search the index of the EMR on the blockchain and locate all the nodes that store the shares of the EMR. In theory, the data user needs to request at least *t* nodes to get the shares, and then, the original EMR can be recovered.

EMRs’ reconstruction: The authorized data users can collect a set of EMR shares $s_{i}$ and then reconstruct the EMR *R* with a specific coefficient matrix $M_{t\times \tau }^{-1}$, which will be discussed in Section 4.

(2) $${\left(x_{1},x_{2},\ldots ,x_{t}\right)}^{T}=M_{t\times \tau }^{-1}\cdot {\left(s_{k_{1}},s_{k_{2}},\ldots ,s_{k_{\tau }}\right)}^{T}$$

Here, τ(t≤τ≤n) EMR shares $\left\{s_{k_{1}},s_{k_{2}},\ldots ,s_{k_{\tau }}\right\}$$\subseteq \left\{s_{1},s_{2},\ldots ,s_{n}\right\}\;\left(k_{\tau }\in \left\{1,2,\ldots ,n\right\}\right)$ can reconstruct the original subsections, $\left\{x_{1},x_{2},\ldots ,x_{t}\right\}$, of the EMR even if a few shares are tampered with or discarded. This efficient data reconstruction process can not only make the message sharing scheme more lightweight, but also improve the efficiency of the verifying and recovering processes. A theoretical analysis of this message sharing scheme is shown in Section 4.

EMRs’ decoding. When the subsections, $\left\{x_{1},x_{2},\ldots ,x_{t}\right\}$, of the EMR have been reconstructed, the recovered subsections of the EMR will be decoded by the interleaving decoder and the original EMR *R* obtained. After that, these recovered EMRs can be processed by authorized consumers with different purposes. Apparently, the EMRs stored in the healthcare blockchain system can be used by not only the patients and the medical doctors, but also the insurance companies, researchers, and others.

In addition, in order to improve efficiency, the processes of EMRs’ interleaving encoding and construction and EMRs’ reconstruction and interleaving decoding can be embedded into the smart contract [29]. This computer trading agreement can prevent the malicious users or adversaries from destroying the EMRs. In addition, blockchain technology makes the EMRs’ data more transparent and credible. Each EMR serves as a transaction that can be recorded into the healthcare blockchain system, which can be verified by the universal verifiable or end-to-end verifiable open blockchain audit trail. Neither the shares’ data processing in the healthcare blockchain system, nor the smart contract are within the scope of this paper, and we will devote ourselves to the security proof and performance evaluation of our proposed scheme in the following sections.

## 4. Security Proof and Analysis

In this paper, we assumed that the shares were encrypted before being transmitted in the blockchain system and that any proper secret negotiation algorithm could be employed to generate the secret keys. The adversary wants to access the EMRs without authorization. Apparently, the adversary can obtain all the private information about the patients and healthcare institutions once the EMRs are leaked. To get an EMR, the adversary needs to capture the shares transmitted in the network. In the following, we first analyze the correctness of the proposed privacy protection scheme. In the healthcare blockchain system, the patient and medical doctor have the rights to create the EMRs, but the unauthorized user is not allowed to join this system. As the original EMRs have been encoded with a special method and sent to many mining nodes in the form of different shares, we prove that the adversary who intercepts no more than *t* shares cannot recover the original EMRs. Even if the adversary obtains all the information of original EMRs, he/she cannot tamper with it without knowing the rule of the interleaving encoder. After that, the EMRs can be correctly verified and recorded by the mining nodes. If one mining node attempts to tamper with the shares, the malicious behavior will be discovered, because it cannot pass the verification of other mining nodes in the blockchain. Therefore, the proposed privacy protection scheme is correct, and the valid EMRs will be correctly collected and recorded in the blockchain.

The security analysis of the proposed privacy protection scheme is presented as follows. Here, we mainly prove that the (t,n)-threshold lightweight message sharing scheme is secure as shown in Theorems 1 and 2.

**Theorem** **1.** *Any τ(t≤τ≤n) EMR shares $\{s_{k_{1}},s_{k_{2}},\ldots ,s_{k_{\tau }}\}\subseteq \left\{s_{1},s_{2},\ldots ,s_{n}\right\}\;\left(k_{\tau }\in \left\{1,2,\ldots ,n\right\}\right)$ can recover the integrated information of the original EMR R.*

**Proof.**  We consider the worst case that the least *t* shares can recover the EMR and take the following two cases as the proof of Theorem 1.

Case 1: In this case, we consider that the first *t* EMR shares $\left\{s_{1},s_{2},\ldots ,s_{t}\right\}$ constructed by Equation (1) can recover the integrated information of EMR $\left\{x_{1},x_{2},\ldots ,x_{t}\right\}$. The first *t* EMR shares are calculated as follows:

(3) $$\left\{\begin{array}{c} s_{1}=x_{1}+x_{2}+\cdots +x_{t}\;\mathrm{mod}\;p\\ s_{2}=s_{1}+2x_{2}+\cdots +x_{t}\;\mathrm{mod}\;p\\ \;\vdots \\ s_{t}=s_{1}+s_{2}+\cdots +tx_{t}\;\mathrm{mod}\;p \end{array}\right.$$

We can present Equation (3) in a matrix form, which is shown in Equation (4):

(4) $${\left(s_{1},\ldots ,s_{i},\ldots ,s_{t}\right)}^{T}=M_{1}\cdot {\left(x_{1},\ldots ,x_{i},\ldots ,x_{t}\right)}^{T}$$

where $M_{1}$ is a coefficient matrix, which can be denoted as Equation (5):

(5) $$M_{1}=\left(\begin{array}{ccccc} a_{11} & \cdots & a_{1j} & \cdots & a_{1t}\\ \vdots & \; & \vdots & \; & \vdots \\ a_{i1} & \cdots & a_{ij} & \cdots & a_{it}\\ \vdots & \; & \vdots & \; & \vdots \\ a_{t1} & \cdots & a_{tj} & \cdots & a_{tt} \end{array}\right)$$

Next, according to Equation (3), the coefficients in matrix $M_{1}$ can be generated as shown in Equation (6):

(6) $$a_{ij}=\left\{\begin{array}{c} 2^{i-1}+2^{i-j-1}\left(j-2\right),\;\mathrm{if}\;2\leq j<i,\\ 2^{j-1}+j-1,\;\mathrm{if}\;j=i,\\ 2^{i-1},\;\mathrm{if}\;i<j\leq t,\\ 2^{i-2},\;\mathrm{if}\;j=1,i>1. \end{array}\right.$$

In order to prove that the first *t* EMR shares $\left\{s_{1},s_{2},\ldots ,s_{t}\right\}$ can recover the integrated information of the EMR *R*, we should prove that matrix $M_{1}$ is invertible first. With one meaning, the determinant of $M_{1}$, i.e., $|M_{1}|$, is a non-zero number. Next, we transform the matrix $M_{1}$ with the following Algorithm 1 into a diagonal matrix. Then, we can conclude that the determinant of matrix $M_{1}$ is t!, which is not zero as $|M_{1}|=t!$. Therefore, we can uniquely obtain $\left\{x_{1},x_{2},\ldots ,x_{t}\right\}$ according to Equation (3) when we get the first *t* EMR shares $\left\{s_{1},s_{2},\ldots ,s_{t}\right\}$.
**Algorithm 1** Matrix transform algorithm.**Input:** Square matrix $M_{1}$**Output:** Lower triangular determinant 1:Count the size of $M_{1}$ which is composed of *t* rows and columns 2:**for**i=2 to *t*
**do** 3: $M_{1}\left(i,:\right)=M_{1}\left(i,:\right)-2^{i-1}\cdot M_{1}\left(1,:\right)$ 4:**end for** 5:**for**j=2 to *t*
**do** 6: $M_{1}\left(:,j\right)=-M_{1}\left(:,1\right)+M_{1}\left(:,j\right)$; 7: **for**
k=2 to j−1
**do** 8:  $M_{1}\left(k,:\right)=M_{1}\left(k,:\right)-M_{1}\left(k,j\right)/M_{1}\left(j,j\right)\cdot M_{1}\left(j,:\right)$; 9: **end for**10: **for**
l=j+1 to *t*
**do**11:  $M_{1}\left(l,:\right)=M_{1}\left(l,:\right)-2^{l-j-1}\cdot M_{1}\left(j,:\right)$;12: **end for**13: **end for**

Case 2: We take another situation in which the first i(0≤i<t) congruence equations are chosen from Equation (1); the other t−i congruence equations are obtained from the last n−t equations constructed by Equation (1). The last n−t equations are shown as follows in Equation (7):

(7) $$\left\{\begin{array}{c} s_{t+1}=\frac{s_{1}}{2}+\frac{s_{2}}{3}+\cdots +\frac{s_{t}}{1+t}\;\mathrm{mod}\;p\\ s_{t+2}=\frac{s_{1}}{3}+\frac{s_{2}}{4}+\cdots +\frac{s_{t}}{2+t}\;\mathrm{mod}\;p\\ \;\;\vdots \\ s_{n}=\frac{s_{1}}{n-t+1}+\frac{s_{2}}{n-t+2}+\cdots +\frac{s_{t}}{n-t+t}\;\mathrm{mod}\;p \end{array}\right.$$

Next, we plan to prove that any subset of *t* EMR shares from $\{s_{1},s_{2},\ldots ,s_{t},s_{t+1},\ldots ,s_{n}\}$ is equivalent to the first *t* EMR shares $\left\{s_{1},s_{2},\ldots ,s_{t}\right\}$. Suppose we choose i(0≤i<t) EMR shares: $\left\{s_{k_{1}},s_{k_{2}},\ldots ,s_{k_{i}}\right\}$, $1\leq k_{1}<k_{2}<\cdots <k_{i}\leq t$ from $\left\{s_{1},s_{2},\ldots ,s_{t}\right\}$ and choose t−i EMR shares: $\left\{s_{t+k_{i+1}},s_{t+k_{i+2}},\ldots ,s_{t+k_{t}}\right\}$, $1\leq k_{i+1}<k_{i+2}<\cdots <k_{t}\leq n-t$ from $\left\{s_{t+1},s_{t+2},\ldots ,s_{n}\right\}$. In this case, congruence equations can be described in the matrix form as shown in Equation (8):

(8) $$\left(\begin{array}{c} s_{k_{1}}\\ \vdots \\ s_{k_{i}}\\ s_{t+k_{i+1}}\\ \vdots \\ s_{t+k_{t}} \end{array}\right)=M_{2}\left(\begin{array}{c} s_{1}\\ \vdots \\ s_{i}\\ s_{i+1}\\ \vdots \\ s_{t} \end{array}\right)$$

where $M_{2}$ is a coefficient matrix, which can be denoted as Equation (9):

(9) $$M_{2}=\left(\begin{array}{ccccccc} 0 & \cdots & 1 & \cdots & 0 & \cdots & 0\\ \vdots & \; & \vdots & \; & \vdots & \; & \vdots \\ 0 & \cdots & 0 & \cdots & 1 & \cdots & 0\\ \frac{1}{k_{i+1}+1} & \cdots & \frac{1}{k_{i+1}+k_{1}} & \cdots & \frac{1}{k_{i+1}+k_{i}} & \cdots & \frac{1}{k_{i+1}+t}\\ \vdots & \; & \vdots & \; & \vdots & \; & \vdots \\ \frac{1}{k_{t}+1} & \cdots & \frac{1}{k_{t}+k_{1}} & \cdots & \frac{1}{k_{t}+k_{i}} & \cdots & \frac{1}{k_{t}+t} \end{array}\right)$$

Now, we need to prove that the equations constructed by $\left\{s_{k_{1}},s_{k_{2}},\ldots ,s_{k_{i}},s_{t+k_{i+1}},\ldots ,s_{t+k_{t}}\right\}$ are equivalent to those constructed by $\left\{s_{1},s_{2},\ldots ,s_{t}\right\}$. In other words, these two equation sets should have the same solution. We can calculate the determinant of $M_{2}$ as shown in Equation (10):

(10) $$\begin{array}{cc} |M_{2}|= & {\left(-1\right)}^{1+\cdots +i+k_{1}+\cdots +k_{i}}\\ \; & \cdot \frac{\prod_{Q<W;Q,W\in \left[1,t\right]\backslash \left\{k_{1},\ldots ,k_{i}\right\}}\;\;\;\;\;\;\;\;\;\;\;\;\;\;\;\;\;\left(W\;\;-\;\;Q\right)\cdot \prod_{i+1\leq v<u\leq t}\;\;\left(k_{u}\;\;-\;\;k_{v}\right)}{\prod_{i+1\leq u\leq t,W\in \left[1,t\right]\backslash \left\{k_{1},\ldots ,k_{i}\right\}}\;\;\;\;\;\;\;\;\;\;\;\;\;\;\;\;\;\left(k_{u}-W\right)} \end{array}$$

Therefore, we can derive that the matrix $M_{2}$ is invertible since the determinant of $M_{2}$ is a non-zero number. The first *t* EMR shares $\left\{s_{1},s_{2},\ldots ,s_{t}\right\}$ can be linearly expressed by $\left\{s_{t+k_{i+1}},s_{t+k_{i+2}},\ldots ,s_{t+k_{t}}\right\}$. Next, based on the proof of Case 1, we can derive that there should be a unique solution for the congruence equations in Case 2. □

In fact, we can rewrite the congruence Equation (1) in the matrix form as follows:

(11) $${\left(s_{1},\ldots ,s_{t},s_{t+1},\ldots ,s_{n}\right)}^{T}=M_{n\times t}\cdot {\left(x_{1},x_{2}\ldots ,x_{t}\right)}^{T}$$

Any subset of *t* EMR shares from $\left\{s_{1},s_{2},\ldots ,s_{n}\right\}$ corresponds to *t* rows of the matrix *M*. According to Case 1, we derive that the EMR information $\left\{x_{1},x_{2},\ldots ,x_{t}\right\}$ can be uniquely recovered from the first *t* EMR shares $\left\{s_{1},s_{2},\ldots ,s_{t}\right\}$. According to Case 2, we derive that any subset of *t* EMR shares is equivalent to the first *t* EMR shares $\left\{s_{1},s_{2},\ldots ,s_{t}\right\}$. Combining Case 1 and Case 2, we can derive that any *t* rows of the matrix *M* are linearly independent and any *t* EMR shares can decide the EMR information $\left\{x_{1},x_{2},\ldots ,x_{t}\right\}$. Further, the original EMR *R* is reconstructed successfully by an interleaving decoder. This completes the proof of Theorem 1.

Based on Theorem 1, we can guarantee that it can recover the original EMR *R* with only *t* shares without obtaining all the *n* shares. Even if a few shares have been destroyed by the system problem, this does not affect the reconstruction of the original EMRs. Therefore, our proposed scheme can greatly improve the fault-tolerant capability of the healthcare blockchain system, which will be shown in the following performance evaluation section.

Next, we analyze the security of the message sharing scheme and prove its security in the other situation. In the healthcare blockchain system, the adversary may eavesdrop and decrypt the shares. However, we can prove that even if the adversary successfully decrypts a set of the shares, they cannot recover the original EMR *R* in Theorem 2.

**Theorem** **2.** *Any $t^{\prime }\left(t^{\prime }<t\right)$ EMR shares of each EMR R cannot recover the integrated information of the EMR R.*

**Proof.**  Suppose that the malicious adversary can successfully obtain $t^{\prime }\left(t^{\prime }<t\right)$ EMRs shares $\{s_{1}^{\prime },s_{2}^{\prime },\ldots ,s_{t^{\prime }}^{\prime }\}\subseteq \left\{s_{1},s_{1},\ldots ,s_{n}\right\}$. According to Equation (1), the malicious adversary can rebuild a set of congruence equations with *t* variables $\left\{x_{1},x_{2},\ldots ,x_{t}\right\}$ as follows:

(12) $$A=M_{t^{\prime }\times t}B$$

Here, $A={\left(s_{1}^{\prime },s_{2}^{\prime },\ldots ,s_{t^{\prime }}^{\prime }\right)}^{T}$ and $B={\left(x_{1},x_{2},\ldots ,x_{t}\right)}^{T}$. The $M_{t^{\prime }\times t}$ is a matrix over a field *F*. Let $F_{p}$ be a field and *M* be a matrix over $F_{p}$. In consideration of the augmented matrix of $M_{t^{\prime }\times t}$, ${\left(M|A\right)}_{t^{\prime }\times \left(t+1\right)}$, we can derive that ${\left(M|A\right)}_{t^{\prime }\times \left(t+1\right)}=M_{t^{\prime }\times t}A$. Depending on the ranks of $M_{t^{\prime }\times t}$ and *A*, Theorem 2 can be proven by two cases as follows:

Case 1: The rank of matrix $M_{t^{\prime }\times t}$ is not much more than that of matrix ${\left(M|A\right)}_{t^{\prime }\times \left(t+1\right)}$. In this case, there is no solution for the equation set, and the integrated information of EMR *R* cannot be recovered by the malicious adversary. It is more likely to happen once the malicious adversary obtains the wrong number of EMR shares $\left\{s_{1}^{\prime },s_{2}^{\prime },\ldots ,s_{t^{\prime }}^{\prime }\right\}$.

Case 2: The rank of matrix $M_{t^{\prime }\times t}$ is the same as the rank of matrix ${\left(M|A\right)}_{t^{\prime }\times \left(t+1\right)}$. In this case, there exist $t^{\prime }$ equations, but *t* variables. Hence, the malicious adversary cannot recover the integrated information of the EMR *R*. Then, we consider the worst case that the malicious adversary can obtain $t^{\prime }=t-1$ EMR shares. Unfortunately, the malicious adversary can only obtain $|F_{p}|$ lawful solutions.

From the above Cases 1 and 2, we can derive that the malicious adversary cannot recover integrated information of the EMR *R* by $t^{\prime }\left(t^{\prime }<t\right)$ EMRs shares in a large-sized field. This completes the proof of Theorem 2. □

Now that Theorems 1 and 2 prove that only τ(t≤τ≤n) shares can successfully recover the integrated information of EMRs. In our proposed scheme, the EMRs that contain the sensitive information of patient and medical institution were split and reconstructed. Even though the adversary collects part of the (less than *t*) shares, he/she cannot recover the integrated original EMRs. Even worse, he/she obtains more (no less than *t*) shares, and he/she cannot obtain any information since he/she does not know the principle of the interleaving coder. Consequently, this scheme not only can ensure the data security, but also can protect the privacy of the patient and medical institution.

## 5. Performance Evaluation and Analysis

In this section, we give the performance evaluation and analysis of the proposed scheme with existing schemes. We implemented our scheme on a strong server with the Windows operation system, 128 G memory, and 2 T external storage. In total, seven blockchain nodes were simulated on the server. Here, we simulated the message sharing scheme in the healthcare blockchain system with the parameters that are shown in Table 2. We assumed that 5000 EMRs of general users were employed for this performance evaluation. A new block was constructed when 10 new EMRs were generated. We chose three EMR sharing schemes with different encryption methods for the comparison, i.e., the scheme in [7], which was designed based on blockchain-enabled health information exchange networks, the cloud-based scheme in [11]. and the IoT-based scheme in [12], which were designed based on the centralized healthcare service system. These three schemes dealt with the complete EMRs, but our proposed scheme was performed with short shares in the healthcare blockchain system. Though some other schemes also exist in the literature, we did not take them into consideration in this section because either they had worse performance or it was unfair to compare them with our scheme. Then, the performances in terms of the energy consumption, storage space, and network fault tolerance of our scheme and the three similar schemes are given below. Moreover, we analyze the security against the latency for the proposed scheme in the healthcare blockchain system in Section 5.4.

### 5.1. Energy Consumption

**Figure 4:**
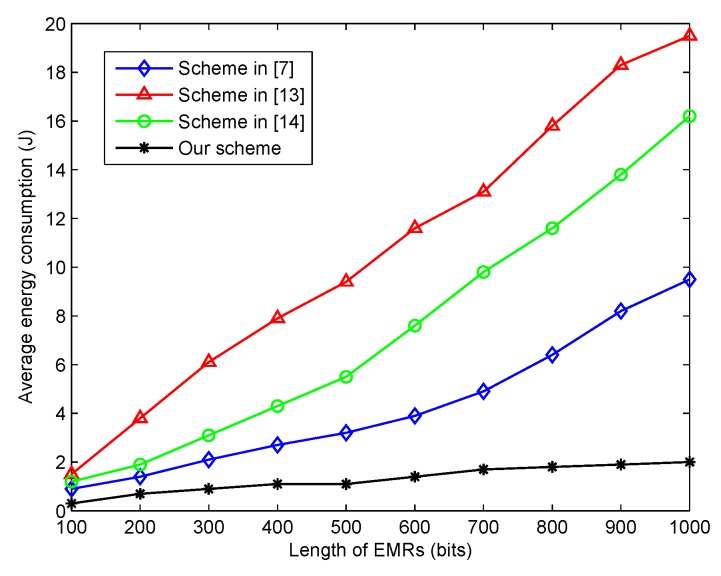
The average energy consumption for different lengths of EMRs.

In this phase, we made an average energy consumption comparison between the proposed scheme and the three other schemes as shown in Figure 4. For one EMR in the healthcare blockchain system, it would be processed in four steps, such as the EMR’s share construction, the EMR’s share verification and confirmation, the EMR’s share retrieval, and the EMR’s share reconstruction. As in the steps of EMR share construction and reconstruction, the computing complexity should be considered since the energy was mainly consumed by the computing processes. Here, the original EMRs was divided into *t* pieces by the interleaving encoder and constructed into *n* shares, and t(t−2)+(n−t)(t−1) add operations, (n−t−1)(t−1) multiply operations, and *n* modulo operations were needed for one EMR. These operations were more efficient than the exponentiation and bilinear pairing operations in the majority of message sharing schemes. The divided share was significantly smaller than the original EMRs to be processed with less energy consumption.

In addition, the energy consumption for the delivery of the EMRs shares in the healthcare blockchain system should be given more consideration. We first assumed that the size of one EMRs was *l* bits. In the proposed scheme, each share only had l/t bits, and hence, only (l/t)×n×(m−1) bits of data for one node needed to be verified and confirmed. However, in the other three schemes, at least l×n×(m−1) needed to be verified and confirmed in the network. Therefore, as the black line shows in Figure 4, the proposed scheme had more energy savings than the three other schemes from the literature.

### 5.2. Storage Space Efficiency

**Figure 5:**
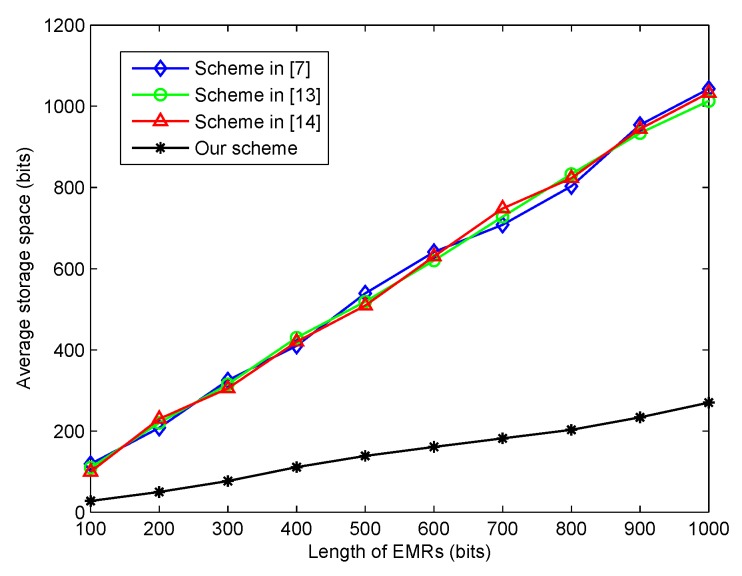
The average storage space for different lengths of EMRs.

In general, the block in the blockchain had a limited size. Hence, the size of the transaction would influence the efficiency of the transaction implementation. In this section, we evaluated the average storage space for each transaction in the proposed scheme. As the original EMRs were divided into *n* shares, each share served as one transaction, which would be verified and recorded in the blockchain. As shown in Figure 5, the simulation results indicated that the average storage space needed linearly increased with the length of the EMRs. Note that the necessary storage space needed contained not only the length of the EMRs, but some extra basic information. However, with the application of the lightweight (t,n)-threshold message sharing scheme, the average storage space needed with one share was significantly smaller than that of the three other schemes with the whole length of the EMRs.

### 5.3. Network Fault Tolerance

**Figure 6:**
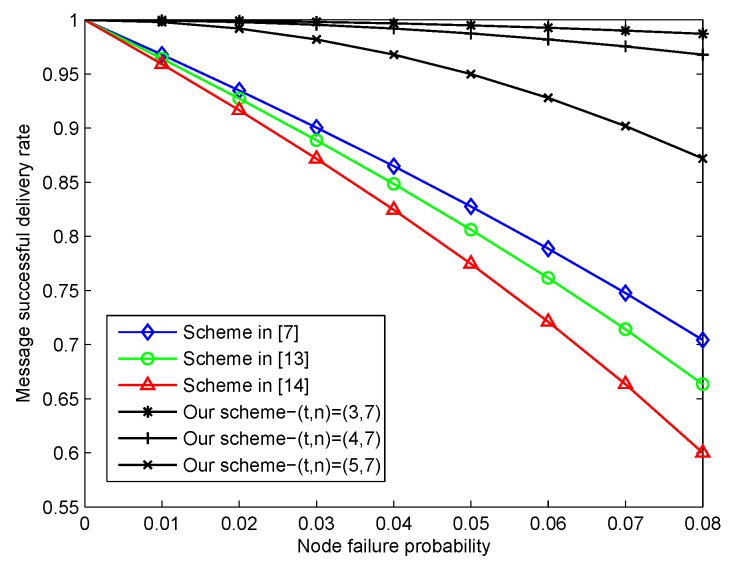
The successful message delivery rate with the node failure probability.

The network fault tolerance depicted the stability of the healthcare blockchain system, and the message sharing scheme could improve the ability of the network fault tolerance. In this part, we considered the successful message delivery rate with respect to the node failure probability among the similar literature schemes and our proposed scheme with different (t,n)={(3,7),(4,7),(5,7)}, which are shown in Figure 6. Our proposed scheme always outperformed the three other schemes with the node failure probability ranging from zero to 0.08. When t=3,4, the successful message delivery rate of our scheme was more than 0.97. In the worst case of a node failure probability of 0.08, the success rate of our scheme (t,n)=(5,7) was 0.87, which was greater than the three other schemes with the success rate approximately being 0.6 to 0.7. Therefore, the proposed scheme could significantly improve the reliability of the healthcare blockchain system.

### 5.4. Security Against the Latency

**Figure 7:**
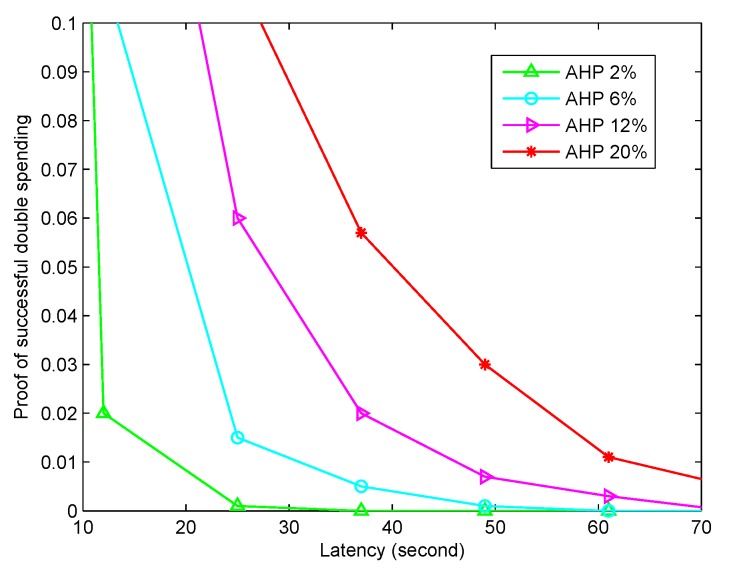
The security against different latencies. Here, we take the impacts from different attacker hash powers (AHP) into consideration.

The theoretical security proof in Section 4 proved that our message sharing scheme was correct and secure, but the distributed healthcare blockchain system equipped with this scheme also could be affected by other latencies. More importantly, the double spending problem should be taken into consideration, which had a big influence on the security of the transaction implementation in the healthcare blockchain system. We assumed that the visitor volume for each block was 50 requests/minute, and the relations between the proof of successful double spending and the latency are shown in Figure 7 with different attacker hash powers (AHP). The simulation results indicated that the network security level could influence the number of transaction confirmations. More confirmations should be processed if the attacker hash power is higher. We hope that the rapid validation and responsiveness will be significant for large volume and high volume healthcare blockchain systems.

## 6. Conclusions

In this paper, we proposed a lightweight privacy-preserving cross-institution EMR sharing scheme based on the blockchain technique and a lightweight (t,n)-threshold message sharing scheme. The interleaving encoding algorithm was employed to destroy the semantic meanings of the original EMRs and hide the sensitive information of the patient and medical institution. The (t,n)-threshold message sharing scheme first constructed the encoded EMRs into *n* shorter shares, and this would improve the efficiency of the data processing. Different from existing blockchains, the shares rather than the original EMRs were stored in the blockchain nodes in a random manner. In the EMR retrieval process, the data users needed to first locate the blockchain nodes that stored the shares of the EMR of interest and requested all the related shares. Then, the original EMR could be reconstructed with at least t(1<t≤n)) shares. This scheme could not only protect the data security, but also improve the efficiency of data sharing between institutions and data users. Moreover, we performed a series of experiments to evaluate the performance of the proposed scheme, and the simulation results showed that it significantly decreased the energy consumption and storage space compared to existing schemes.

Our scheme could be further improved in several aspects. First, we will make an effort to design a more lightweight message sharing scheme to improve the efficiency of the EMRs data processing in our future work. Second, we will research the combination of blockchain and mobile edge computation in efficient healthcare service systems with the explosive increase of data terminals. Third, our scheme did not provide an efficient EMR retrieval mechanism; hence, we will design a novel index structure for the shares of EMRs. This could greatly improve the experience of both the data users and healthcare institutions.

## Figures and Tables

**Table 1:** Main terms of the lightweight privacy-preserving mechanism.

Terms	Description
User	The entities who create the original EMRs.
Original EMRs	The original diagnosis records *R* created by the user.
Sub-messages	The encrypted EMRs $\left\{x_{1},x_{2},\ldots ,x_{t}\right\}\;\left(1<t\leq n\right)$ encoded by interleaving encoder. Here, *n* is the shares described as follows.
Shares	The EMR shares constructed from sub-messages. $\left\{s_{1},s_{2},\ldots ,s_{n}\right\}$ will be stored in the blockchain network, and $\left\{s_{k_{1}},s_{k_{2}},\ldots ,s_{k_{\tau }}\right\}\subseteq \left\{s_{1},s_{2},\ldots ,s_{n}\right\}\;\left(k_{\tau }\in \left\{1,2,\ldots ,n\right\},t\leq \tau \leq n\right)$ are collected and used for EMRs’ reconstruction.
Blockchain Network	The system that verifies and stores the EMR shares and provides a platform for the cross-institutional sharing of EMRs.
Smart Contract	The processes of EMRs’ creation and storage and EMRs’ extraction and use have been embedded into the automatic smart contract, which cannot be affected by malicious behaviors.
Recovered EMRs	The integrated EMRs decoded from the reconstructed sub-messages, which can be used for diagnostic reference and research.
Consumer	The entities that use the recovered EMRs data.

**Table 2:** Simulation parameters.

Parameter	Value
Average block time	12 s
Size of EMRs	100, 200, …, 1000 bits
(t,n)	(4,7)
Number of nodes	100
Number of the users	5000

## References

[1] Sun J.T., Fang Y.G. (2010). Cross-domain data sharing in distributed electronic health record systems. IEEE Trans. Parallel Distrib. Syst..

[2] Tien J.M., Goldschmidt-Clermont P.J. (2009). Healthcare: A complex service system. J. Syst. Sci. Syst. Eng..

[3] Li C.J., Liu L., Chen S.Z., Wu C.C., Huang C.H., Chen X.M. Mobile healthcare service system using RFID. Proceedings of the IEEE International Conference on Networking, Sensing and Control.

[4] Nakamoto S. (2008). Bitcoin: A Peer-To-Peer Electronic Cash System. https://bitcoin.org/bitcoin.pdf.

[5] Gervais A., Karame G.O., Wust K., Glykantzis V., Ritzdorf H., Capkun S. On the Security and Performance of Proof of Work Blockchains. Proceedings of the 2016 ACM SIGSAC Conference on Computer and Communications Security.

[6] Dubovitskaya A., Xu Z.G., Ryu S., Schumacher M., Wang F.S. (2017). Secure and trustable electronic medical records sharing using blockchain. Am. Med. Inform. Assoc. Annu. Symp. Proc..

[7] Peterson K., Deeduvanu R., Kanjamala P., Boles K. A Blockchain-Based Approach to Health Information Exchange Networks. In Proceeding of NIST Workshop Blockchain Healthcare.

[8] Ge Y.R., Ahn D.K., Unde B., Gage H.D., Carr J.J. (2013). Patient-controlled sharing of medical imaging data across unaffliated healthcare organizations. J. Am. Med. Inform. Assoc..

[9] Vest J.R., Gamm L.D. (2010). Health Information Exchange: persistent challenges and new strategies. J. Am. Med. Inform. Assoc..

[10] Gordon W.J., Catalini C. (2018). Blockchain technology for healthcare: Facilitating the transition to patient-driven interoperability. Comput. Struct. Biotechnol. J..

[11] Benjamin F., Ermakova T., Junghanns P. (2015). Collaborative and secure sharing of healthcare data in multi-clouds. Inf. Syst..

[12] Yang Y., Zheng X.H., Guo W.Z., Liu X.M., Chang V. (2019). Privacy-preserving smart IoT-based healthcare big data storage and self-adaptive access control system. Inf. Sci..

[13] Azaria A., Ekblaw A., Vieira T., Lippman A. Medrec: Using blockchain for medical data access and permission management. Proceedings of the 2016 2nd International Conference on Open and Big Data (OBD).

[14] Yue X., Wang H., Jin D., Li M., Jiang W. (2016). Healthcare data gateways: Found healthcare intelligence on blockchain with novel privacy risk control. J. Med. Syst..

[15] Johnson D., Menezes A., Vanstone S. (2001). The elliptic curve digital signature algorithm (ECDSA). Int. J. Inf. Secur..

[16] Li C.Y., Chen X., Chen Y.L., Hou Y.Y., Li J. (2019). A New Lattice-based Signature Scheme in Post-Quantum Blockchain Network. IEEE Access.

[17] Miguel C., Liskov B. (1999). Practical Byzantine Fault Tolerance.

[18] Irving G., Holden J. (2016). How blockchain-timestamped protocols could improve the trustworthiness of medical science. F1000Research.

[19] Taylor P. Applying Blockchain Technology to Medicine Traceability. https://www.securingindustry.com/pharmaceuticals/applying-blockchain-technology-to-medicine-traceability/s40/a2766/#.XoBiF7h5tPb.

[20] Witchey N.J. (2015). Healthcare Transaction Validation via Blockchain Proof-Of-Work, Systems and Methods. U.S. Patent.

[21] Mettler M. Blockchain technology in healthcare: The revolution starts here. Proceedings of the 2016 IEEE 18th International Conference on e-Health Networking, Applications and Services (Healthcom).

[22] Chen L.X., Lee W.K., Chang C.C., Choo K.R., Zhang N. (2019). Blockchain based Searchable Encryption for Electronic Health Record Sharing. Futer Gener. Comput. Syst..

[23] Amos B. (2011). Secret-sharing schemes: A survey. International Conference on Coding and Cryptology.

[24] Shamir A. (1979). How to share a secret. Commun. ACM.

[25] Bishop A., Pastro V., Rajaraman R., Wichs D. (2016). Essentially optimal robust secret sharing with maximal corruptions. Annual International Conference on the Theory and Applications of Cryptographic Techniques.

[26] Yan X., Liu X., Yang C.N. (2018). An enhanced threshold visual secret sharing based on random grids. J. Real-Time Image Process..

[27] Pang L.J., Wang Y.M. (2005). A new (t, n) multi-secret sharing scheme based on Shamir’s secret sharing. Appl. Math. Comput..

[28] Wang N., Fu J.S., Zeng J.W., Bhargava B.K. (2018). Source-location privacy full protection in wireless sensor networks. Inf. Sci..

[29] Shen W.T., Qin J., Tu J., Hao R., Hu J.K. (2019). Enabling identity-based integrity auditing and data sharing with sensitive information hiding for secure cloud storage. IEEE Trans. Inf. Forensics Secur..

[30] Crosby M., Pattanayak P., Verma S., Kalyanaraman V. (2016). Blockchain technology: Beyond bitcoin. Appl. Innov..

